# Streamlining counterfactual ecosystem restoration and conservation impact evaluation in R with the spatialBACI package

**DOI:** 10.3897/BDJ.14.e182671

**Published:** 2026-05-27

**Authors:** Jasper Van doninck, Wietske Bijker, Louise Willemen

**Affiliations:** 1 Faculty of Geoinformation Science and Earth Observation (ITC), University of Twente, Enschede, Netherlands Faculty of Geoinformation Science and Earth Observation (ITC), University of Twente Enschede Netherlands

**Keywords:** before-after-control-impact, earth observation, geospatial data, conservation, restoration, R package

## Abstract

Before-after-control-impact (BACI) assessments are crucial in determining effectiveness of ecosystem conservation and restoration actions. This type of analysis can strongly benefit from earth observation and other geospatial datasets. The R package *spatialBACI* provides functionalities to streamline use of spatial datasets in some of the main steps of BACI assessment for ecosystem conservation: defining impact and control units, matching impact and control units and impact assessment. We demonstrate the utility of the package for a restoration site.

## Introduction

Measuring the impact of nature conservation and restoration interventions is crucial for adaptive management, to learn from past effort and improve future conservation and restoration effectiveness. While monitoring of a site after conservation or restoration interventions can provide valuable insights, it can by itself typically not be used to infer causal impact (Baylis et al. 2016). Counterfactual assessment can assist in establishing this causality. In the context of conservation or restoration effectiveness monitoring, counterfactual analysis attempts to establish the difference between the (intended or unintended) outcomes of an intervention and the outcomes if no action had been taken (Coetzee and Gaston 2021). In the absence of the random implementation of conservation and restoration actions, which is typically not feasible nor desirable, the use of counterfactuals provides the most robust way to assess the impact of conservation and restoration actions (Ribas et al. 2020). The strongest impact evaluation designs are those where outcomes before and after a conservation or restoration intervention are examined for the impact site (also referred to as treatment or intervention) and a control site in a before-after-control-impact (BACI) analysis (Wauchope et al. 2021). Control sites should be environmentally similar to the impact sites to avoid overestimation of conservation or restoration interventions (Andam et al. 2008). The pairing of impact sites with counterfactual control sites is typically done through some form of statistical matching (Schleicher et al. 2020).

Satellite earth observation (EO) and other geospatial datasets can play an important role in counterfactual impact assessment of environmental interventions. Since a culture of counterfactual evaluation is not yet fully embedded in the conservation/restoration community (Coetzee and Gaston 2021), many interventions are not designed with a counterfactual evaluation in mind. As a result, counterfactual in situ data on conservation or restoration outcomes in space and/or time are often lacking. Global coverage over long time series of several satellite missions can act as an alternative in this case (del Río-Mena et al. 2021). Furthermore, EO and other geospatial datasets are critical to link impact sites to their environmentally similar counterfactuals.

Unfortunately, access to geospatial data and processing can be a barrier to users without a solid background in these domains. With this R package, we therefore aim to streamline the use of geospatial datasets into spatially explicit conservation and restoration impact assessment. The focus of the package lies on three main parts of spatial impact assessment: defining impact and control units, matching impact and control units based on geospatial data layers of environmental covariates and impact evaluation through a BACI metric.

## Installation

The *spatialBACI* package can be installed from GitHub using the *devtools* package:

*library(devtools)*

*install_github("Space4Restoration/spatialBACI", dependencies=TRUE)*

## Usage

The package builds on existing R packages including *terra* (Hijmans 2025) and *gdalcubes* (Appel and Pebesma 2019) for geospatial data processing, *data.table* (Barrett et al. 2025) for tabular data processing and *MatchIt* (Ho et al. 2011) for control-impact matching. The idea behind the package is to provide users with an accessible solution that integrates the various aspects of spatial impact assessment, without requiring extensive knowledge on geospatial data processing or control-impact matching. At the same time, we aimed for a flexible and customisable framework where users can choose to rely on open EO and geospatial data only or introduce their own (in situ) datasets or proprietary datasets at several stages in the process. The package should facilitate comparing differences in impact evaluation results from different design parameters.

**Figure 1.**
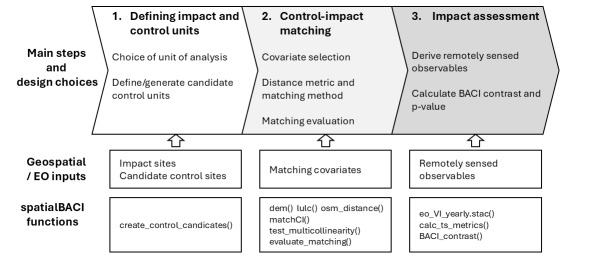
Main steps of counterfactual impact assessment supported by *spatialBACI*.

In the following subsection, we describe the package implementation of three main steps of counterfactual analysis (Fig. 1): 1) defining impact and control units; 2) matching of impact and control units and 3) impact assessment using the matched impact and control units. In each subsection, we first give a brief background on the importance of the step and discuss existing practices, followed by a description of relevant the package functions. Finally, we use the Baviaanskloof dataset included in the package to demonstrate the functioning of the package.

The Baviaanskloof data can be loaded as a *terra* SpatVector object as:

*library(spatialBACI)*

*data(baviaanskloof)*

*library(terra)*

*baviaanskloof = unwrap(baviaanskloof)*

**Figure 2.**
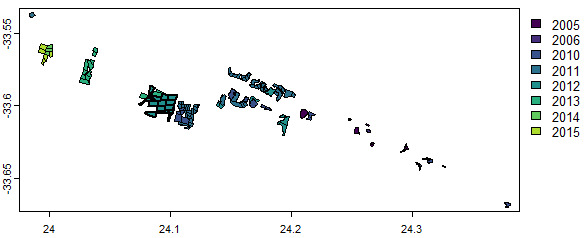
Revegetation year for the Baviaanskloof restoration sites.

The Baviaanskloof dataset (del Río-Mena et al. 2021) contains the sites of large-scale *Portulacaria afra* revegetation in the semi-arid Baviaanskloof Hartland Bawarea Conservancy, South-Africa. Vector attributes indicate in which year the re-vegetation was undertaken by the restoration organisations active in the area (Commonland, Living Land, Grounded) and whether livestock grazing exclusion was implemented as a restoration activity. In our example, we select the sites re-vegetated in 2012 that were not subject to livestock grazing exclusion (see Fig. 2 for plot results):

*impact_sites = baviaanskloof[baviaanskloof$Planting_date==2012 & baviaanskloof$Lifestock_exclusion==0]*

*other_sites = baviaanskloof[baviaanskloof$Planting_date!=2012 | baviaanskloof$Lifestock_exclusion==1]*

*plot(baviaanskloof, "Planting_date")*

*lines(impact_sites, lwd=2)*

### Defining impact and control units

A first critical design consideration in counterfactual impact evaluation is the definition of the spatial unit and scale of analysis (Baylis et al. 2016, Schleicher et al. 2020). This definition of these impact and control units must be informed by a clear theory of change and real world complexity and account for potential positive or negative spillover effects (Baylis et al. 2016, Schleicher et al. 2020).

The unit of analysis can be of different formats, such as points, polygons and rasters. Impact and control units are often explicitly delineated as study site polygons when impact evaluation relies on *in situ* observables such as species counts. This is especially so for standardised monitoring efforts. For example, Wauchope et al. (2022) assessed protected area impact on waterbird populations observed at systematically monitored sites and Terraube et al. (2020) used data from a systematic wildlife count scheme to assess carnivore conservation effectiveness. Impact and control units are then typically defined as those *in situ* site polygons or points within or outside the intervention of interest (e.g. protected area programme), respectively. When impact evaluation relies on EO-derived observables, the definition of control sites is no longer restricted by the availability of *in situ* data, which offers more flexibility in the definition of control sites. For example, Meroni et al. (2017) compared explicitly delineated polygons of land restoration intervention sites (impact) with control polygons generated, based on a set of criteria including size and fractional land cover, and van der Vliet et al. (2024) defined control sites for restoration interventions by random selection, as buffers and through expert knowledge.

Integrating remotely sensed observables in counterfactual impact assessment also makes it possible to define impact and control units as pixels. In that case, pixels within impact areas are compared to control pixels outside the impact area. For example, Jones and Lewis (2015) obtained impact and control units from a random sample of Landsat pixels over forests in- and outside Russian protected areas, respectively. The same approach was followed by Eklund et al. (2016) for forest pixels in- and outside of protected areas in Madagascar and by Brodie et al. (2025) for pixels in- and outside of other effective area-based conservation measures (OECMs) in Colombia, South Africa and the Philippines. Similarly, del Río-Mena et al. (2021) identified control units as pixels with similar vegetation characteristics in the vicinity of the impact pixels within thicket and shrubland restoration intervention sites in South Africa.

For analysis with the pixel as the spatial unit of analysis, we implemented the *create_control_candidates()* function that provides algorithmic generation of (candidate) control units, based on user inputs. This function requires, as its main input, the spatial delineation of the impact site (or sites) as a SpatVector polygon object. Impact units of analysis are then defined as the pixels within the SpatVector object, with spatial resolution and coordinate reference system (CRS) defined by the user. Control units can be defined using a neighbourhood around the impact units, a SpatVector polygon identifying the area from which to select control units, a polygon to exclude as control pixels or a combination of these. Finally, both impact and control units can be further restricted using an inner and/or outer buffer around the impact polygon(s). Excluding areas in the proximity of impact sites as control units reduces the risk that the analysis is affected by leakage or spillover effects. The function returns a raster in which impact pixels have value "1", control pixels have value "0" and pixels excluded as impact or control have a "no data" value.

Using the Baviaanskloof dataset introduced before, impact and control pixels of 60-m resolution can be generated as:

*impact_control_cands = create_control_candidates(impact=impact_sites, resolution=60, control_from_buffer=5000, control_exclude=other_sites, exclude_impact_buffer=300, round_coords=-2, sample_control=0.5)*

*plot(impact_control_cands, type="classes", levels=c("0 - Cand. control", "1 - Impact"))*

**Figure 3.**
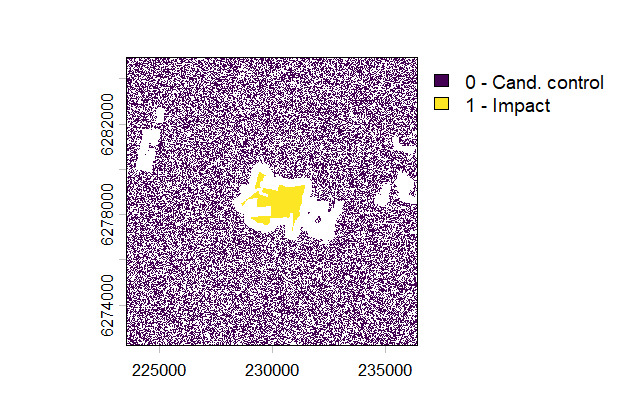
Impact (1), candidate control (0) and excluded pixels (masked) for the Baviaanskloof example.

We here defined that control units are all the 60-m pixels (argument *resolution*) in a buffer of 5000 m (argument *control_from_buffer*) around the selected restoration sites (argument *impact*), with the exception of pixels within other restoration sites (argument *control_exclude*). We also exclude pixels in a buffer of 300 m around the restoration site to account for potential misregistration or spillover effects (argument *excluded_impact_buffer*). Setting the *round_coords* argument to -2 sets the origin of the output SpatRaster to the closest 100 m and the CRS of the output raster is set by default as the UTM zone corresponding to the centroid of the *impact* SpatVector object. Setting the *sample_control* argument to 0.5 results in a random sampling of 50% of the candidate control pixels, which can be useful to reduce computational load for large study areas. Similarly, the *sample_impact* argument can be used to reduce the number of impact units. The *plot()* command returns a visual representation of impact, candidate control and excluded pixels (Fig. 3).

### Control-impact matching

Inappropriate selection of control units may lead to biased estimates of intervention effectiveness (Andam et al. 2008). Conservation and restoration actions are often implemented at poorly-accessible sites with limited value for other activities. Methods that disregard this selection bias of conservation and restoration actions when identifying control units, called "naïve" methods, may, therefore, severely overestimate intervention effectiveness (Joppa and Pfaff 2009, Ribas et al. 2020, Ribas et al. 2021). Methods that limit the control units to be geographically close to the impact units intend to make impact and control as similar as possible, but this assumption is rarely validated (Ribas et al. 2020). Conversely, the goal of statistical matching is to obtain control units environmentally similar to impact units by eliminating the effect of confounders: external variables systematically associated with the allocation of a treatment (i.e. where a conservation or restoration intervention occurs) and the outcome of interest (Ribas et al. 2020, Schleicher et al. 2020). Control-impact matching consists of three main steps: the selection of matching covariates, the selection of distance metric and matching method and the evaluation of the matching results.

#### Covariate selection

While the selection of covariates depends on the application, some general rules apply. Ideally, matching analysis includes a large set of covariates that are likely to impact, directly or indirectly, the selection of the treatment and the outcome of interest (Schleicher et al. 2020, Ribas et al. 2021). Expert knowledge is required to assess these dependencies. In case of doubt, it is advised to include a covariate, as long as only time-invariant variables or variables pre-dating the intervention are used (Stuart 2010, Schleicher et al. 2020). While multicollinearity introduced by adding different variables is often considered to be of minor importance (Ribas et al. 2021), some studies explicitly test for it (e.g. Black and Anthony (2022)).

Common covariates used in control-impact matching to assess conservation or restoration effectiveness include geographic distances to towns, roads or river networks (Andam et al. 2008, Jones and Lewis 2015, Eklund et al. 2016, Terraube et al. 2020), terrain elevation or its derivatives (Jones and Lewis 2015, Eklund et al. 2016, Terraube et al. 2020) or land cover and vegetation characteristics (Eklund et al. 2016, del Río-Mena et al. 2021). Population density (Andam et al. 2008, Terraube et al. 2020), a Human Footprint Index (Brodie et al. 2025) or demographic information (Andam et al. 2008, Terraube et al. 2020) have also been used as direct or indirect indicators of human environmental pressure.

We implemented several functions to facilitate extracting commonly used matching covariates from open geospatial datasets. All these can be used in combination with either the pixel or polygon unit of analysis. Amongst the implemented functions is the *dem()* function which extracts a Digital Elevation Model (DEM) from an online repository and, depending on the provided arguments, calculates terrain slope and aspect. By default, aspect is transformed into northness and eastness, as the circular aspect variable is not suitable for straightforward calculation of environmental similarity. The *osm_distance_roads()* and *osm_distance_places()* functions calculate the geographic distance from each impact or control unit to the nearest road and settlements, respectively, based on OpenStreetMap (OpenStreetMap 2025) data. Finally, the *lulc()* function extracts the land cover classes, by default from the 9-class land cover dataset produced by Impact Observatory, Microsoft and Esri, for the units of analysis.

Building on the *impact_control_cands* raster of impact and control units derived above, elevation and derivatives, distance to roads and land cover covariates can be obtained as:

*dem_match = dem(impact_control_cands)*

*roadsDist_match = osm_distance_roads(impact_control_cands, values="track+")*

*landcover_match = lulc(impact_control_cands, year=2012)*

The different matching covariates can be collated into a format suitable for input to the control-impact matching with the *collate_matching_layers()* function. This function handles the necessary spatial processing of the different spatial input raster, vector or tabular datasets. The function *test_multicollinearity()* plots a correlation matrix of the matching covariates, reports their Variance Inflation Factor (VIF) and allows the user to iteratively identify and remove highly multicollinear (e.g. VIF > 5) covariates :

*matching_input = collate_matching_layers(impact_control_cands, vars_list=list(dem_match, landcover_match, roadsDist_match))*

*matching_input = test_multicollinearity(matching_input)*

#### Distance metric and matching method

Once the matching covariates have been identified and collated, the next step is selecting the distance metric and matching method to match control to impact units. Defining the environmental distance metric allows for calculating the distance matrix used in the matching algorithm. Many distance metrics to describe the similarity between impact and control units exist, each with inherent advantages and disadvantages, so it can be useful to repeat the same analysis with different distance metrics (Schleicher et al. 2020, Ribas et al. 2021). Popular distance metrics in counterfactual analysis for restoration and conservation impact assessment include Mahalanobis distance (Andam et al. 2008, Wauchope et al. 2022) and propensity score distance (Carranza et al. 2014, Terraube et al. 2020, Xiaoyao et al. 2023, Brodie et al. 2025). The propensity score (Rosenbaum and Rubin 2023) combines all selected covariates into a single distance measure that estimates the probability of a unit being submitted to the treatment and can be estimated using statistical approaches, such as logistic or probit models (Ho et al. 2011) with the covariates as independent variables.

After calculating distances metrics, the matching method must be specified. This includes selection of the matching algorithm, ratio of control units per impact unit, replacement and caliper. The matching algorithm determines how impact units are matched with control units given the distance metric. Popular matching algorithms in conservation and restoration evaluation include exact matching (del Río-Mena et al. 2021), nearest neighbour (e.g. Andam et al. (2008), Brodie et al. (2025)) and optimal matching (e.g. D'Alberto et al. (2024)). For most matching algorithms, an impact unit can be matched with either one or several control units. Increasing the number of control units typically increases the accuracy and robustness of post-matching analysis (Ribas et al. 2021), though Del Río-Mena et al. (2023) found that results of subsequent analysis did not significantly change when increasing the number of control units per impact unit from 20 to 100. Replacement is the choice whether or not control units already matched with an impact unit can be retuned to the pool of candidate control units for matching with other impact units. Replacement can reduce the bias in post-matching estimates and helps remove the effect of selection order in some matching algorithms. Replacement is also advantageous when few candidate control units are available relative to the number of impact units (Ribas et al. 2021). Finally, calipers are thresholds that define the maximum difference in the distance metric between impact and control units. Setting calipers (Terraube et al. 2020, Santangeli et al. 2023) restricts the matching to impact and control units that are sufficiently similar, but may result in some impact units lacking matched control units and being excluded from further analysis.

In *spatialBACI*, we implemented the *matchCI()* function to execute the control-impact matching. The function provides a wrapper around the *matchit()* function of the *MatchIt* package and takes as main input the collated matching covariates for the impact and candidate control units (output of *collate_matching_variables()*). The other inputs correspond to those of the *matchit()* function, and control matching parameters such as distance metric, ratio of control units per impact unit and replacement of control units. The function returns a list containing the matching model, the matched control and impact pairs as a data.table object and the spatial reference for the impact/control units.

To demonstrate the *matchCI()* function, we use the collated terrain, land cover and distance to roads covariates. Here, we use ten control pixels per impact pixel (argument *ratio*) with replacement (argument *replace*). All other arguments are set to the default of the *matchit()* function, which implies nearest neighbour matching on the propensity score calculated by logistic regression, without use of a caliper:

*matching_output = matchCI(matching_input, ratio=10, replace=TRUE)*

#### Matching evaluation

It is good practice to evaluate the matching by assessing the similarity of the covariates of impact and control units after matching (Ho et al. 2011). If large differences between impact and control units in the distance metric or matching covariates persist after matching, it may be advisable to repeat matching with a different set of distance metric and matching method. When calipers are used, it should also be evaluated how many impact units were matched and discarded. When replacement of control units is used, it is advised to check the selection rate of matched controls (Schleicher et al. 2020). Furthermore, users may want to test for spatial autocorrelation of the matching output, or assessment of the sensitivity of matching to the presence of unobserved confounders (Schleicher et al. 2020, Black and Anthony 2022).

With the matching model provided as output of *matchCI()*, users have the flexibility to assess the quality of the matching with custom methods. Additionally, we provide some general evaluation of matching results in the *evaluate_matching()* function. This function takes as argument the output of *matchCI()*, together with logical arguments defining whether to plot the covariate overlap before and after matching or a Love plot of the Standardised Mean Difference of the distance metric and matching covariates. Based on this evaluation, users can decide to continue analysis with these matching results or to perform matching with a different set of parameters or covariates.

*evaluate_matching(matching_output)*

### Impact assessment

Once the impact units and control units have been defined, the impact or effectiveness of an intervention can be assessed, based on one or more observables of interest. Conservation and restoration action can have a wide range of desired outcomes and the range of observables or indicators used in impact evaluation therefore can - and often should - also vary widely (Ghoddousi et al. 2022, D'Alberto et al. 2024). While the observables of interest used in conservation or restoration effectiveness studies are often in situ measurements (e.g.Terraube et al. (2020), Wauchope et al. (2022)), remotely sensed observables can be valuable in counterfactual analysis with a BACI design.

#### Remotely-sensed observables

Satellite EO missions acquire data across large spatial extents, often forming long time series. Including EO in impact evaluation, therefore, enables counterfactual frameworks even in the common situation that restoration or conservation actions are not designed with a counterfactual evaluation in mind and counterfactual in situ observables in space and/or time are lacking (Coetzee and Gaston 2021).

Global EO-derived layers of deforestation at yearly or higher intervals are now routinely available (e.g. Hansen et al. (2013)) and have been used for assessing avoided deforestation in protected areas in several studies (e.g. Jones and Lewis (2015), Wendland et al. (2015), Eklund et al. (2016)). Processes of interest in areas under conservation or restoration may often be more gradual and not suitable for representation as binary products such as deforestation layers. For example, van der Vliet et al. (2024) used remotely sensed soil water content, soil temperatures and surface greenness at impact and control sites to monitor restoration effectiveness. EO vegetation indices have also been combined with in situ observation of ecosystems services, after which the EO estimates were tested and used in a BACI framework (del Río-Mena et al. 2020, del Río-Mena et al. 2021).

We implemented several functions in the *spatialBACI* package for access and analysis of free and open satellite EO datasets. These functions build on several other R packages for spatial data querying, access and processing. Data query uses the Spatio-Temporal Image Catalogue (STAC) specification and the R client library implementation in the *rstac* package (Simoes et al. 2021). We use the R package *gdalcubes* (Appel and Pebesma 2019) to create multidimensional data cubes of EO data. This package allows creating proxy data cube objects describing the dimensions of the output cube through lazy evaluation, where data download and processing are only done for the spatial or temporal section of the data cube needed. This is especially useful for analysis where impact and control sites are sparsely distributed over a large geographic extent. We implemented the function *eo_VI_yearly.stac()* to generate yearly vegetation index composite images from a Landsat or Sentinel-2 STAC collection, based on median value compositing. The function requires a STAC endpoint and collection as input, together with the selected vegetation index to calculate and the spatial and temporal specifications of the output data cube.

As an example, we create yearly Landsat NDVI time series with the spatial dimensions of the previously defined SpatRaster of impact and candidate control pixels for the 10-year periods (argument *years*) before and after a re-vegetation intervention in 2012, from images acquired in the months March through May (argument *months*). We specified in the arguments *endpoint* and *collection* that Landsat data are queried from the Planetary Computer STAC catalogue (Microsoft Open Source et al. 2022), on which the implementation of the *eo_VI_yearly.stac()* function focuses. In the argument *maxCloud*, we specify that we only use those Landsat scenes with less than 60% cloud cover. By default, the *eo_VI_yearly.stac()* function masks cloud and cloud shadow, based on the metadata layers provided for the Landsat and Sentinel-2 Level-2 products:

*stac_endpoint = as.endpoint("PlanetaryComputer")*

*stac_collection = as.collection("Landsat", stac_endpoint)*

*vi_before = eo_VI_yearly.stac(impact_control_cands, "NDVI", endpoint = stac_endpoint, collection = stac_collection, years = seq(2012-10, 2012-1, 1), months = 3:5, maxCloud = 60)*

*vi_after = eo_VI_yearly.stac(impact_control_cands, "NDVI", endpoint = stac_endpoint, collection = stac_collection, years = seq(2012+1, 2012+10, 1), months = 3:5, maxCloud = 60)*

Time series of (remotely sensed) observables at impact and control units can be included directly into impact evaluation frameworks (Wauchope et al. 2022). Alternatively, the time series before and after intervention can be summarised into metrics that describe temporal aspects of the time series. *spatialBACI* includes the *calc_ts_metrics()* function that summarises yearly time series in an average and trend component and optionally returns the intercept at the minimum or maximum timestep of the time series. The function requires a SpatRaster or data cube as input and returns an object of the respective class. As an example, the following commands derive average and trend from the 10-year before and after time series obtained above. Adding the *as.SpatRaster()* function converts the resulting objects from a data cube to a SpatRaster object and initiates download and processing of the required imagery:

*avgtrend_before = calc_ts_metrics(vi_before) |> as.SpatRaster()*

*avgtrend_after = calc_ts_metrics(vi_after) |> as.SpatRaster()*

#### BACI contrast and p-value

Finally, the remotely sensed or in situ observables at the impact and matched control units of analysis can be used in a statistical analysis to assess impact. Several methods exist to quantify impact, often using logistic regression on the impact and matched control units (Wauchope et al. 2021). When a single metric represents the condition before and after intervention at impact and control sites, a BACI contrast can be calculated (Meroni et al. 2017, del Río-Mena et al. 2021) to express the difference between sites with and without intervention. The BACI contrast is a difference-in-difference metric expressed as (Meroni et al. 2017):

\begin{varwidth}{50in}\begin{equation*}
            contrast = (\mu_{CA}-\mu_{CB})-(\mu_{IA}-\mu_{IB})
        \end{equation*}\end{varwidth} ,

where *μ* represents the observable of interest and the subscripts C, I, A and B stand for the control and impact unit and after and before period, respectively.

**Figure 4.**
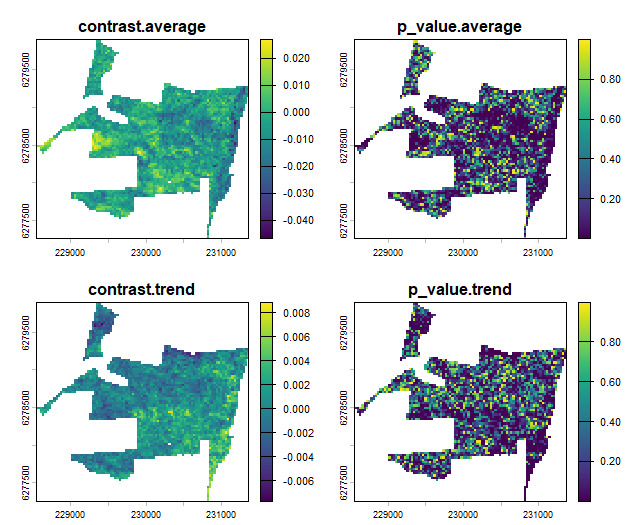
BACI contrast (left) and p-value (right) for NDVI time series average (top) and trend (bottom).

If more than one control unit is used per impact unit or if several impact units and control units are pooled for analysis, a significance testing of the null hypothesis of no impact can be conducted. We implemented a calculation of BACI contrast and p-value of t-test in the *BACI_contrast()* function. This function takes as input a table of the matched control-impact combinations and the spatial layers of the before and after values of the observable(s) to be evaluated. It returns a list containing a data.table object with the BACI contrast and p-value for each provided observable and a spatial object allowing visual inspection of spatial patterns of impact (Fig. 4). Using the different variables created above, we can derive the BACI contrast and impact as:

*baci_results = BACI_contrast(matching_output, before=avgtrend_before, after=avgtrend_after)*

*plot(baci_results$spat)*

## Discussion

In this package, we attempted to implement many aspects of geospatial data processing under the hood. For example, checking coordinate reference systems and projecting is automated where possible. We also attempted to design the different functions used in the impact assessment workflow in such a way that they can be used with different types of units of analysis with minimal changes to syntax; i.e., almost all functions shown here using pixels as spatial units of analysis can also be used to perform counterfactual analysis with the polygons as units of analysis. The standardised processes in this package and the flexibility in unit of analysis facilitates reproduction or impact assessment at different spatial scales. A user can, therefore easily compare the effect of analysis at a regular grid (raster) with geographical (e.g. valley, watershed, landscape) or administrative units of analysis to evaluate sensitivity of results to the choice of scale (Baylis et al. 2016).

Satellite EO programmes generate an enormous amount of data over space and time. It can be challenging to process these EO datasets into a format that can be used in impact evaluation. Yearly time series are a convenient and generally robust, input for impact evaluation frameworks (Wauchope et al. 2021). However, satellite EO datasets typically have a temporal resolution much higher than one year, allowing them to capture seasonal dynamics. In those cases, the choice of how multitemporal data are synthesised can severely impact the outcomes of an EO-supported impact assessment. For example, Del Río-Mena et al. (2023) investigated the importance of time of year for which EO-derived ecosystem services were calculated and observed that restoration impact evaluation outcomes differed, based on this selection of time of year. Furthermore, the "before" and "after" periods in a BACI designs are used to establish the baseline condition and the response to intervention, respectively. However, careful consideration of how these periods are defined is required. Del Río-Mena et al. (2023) noticed that different baseline reference periods in a BACI design could heavily impact evaluation outcomes. Our package enables such sensitivity analysis by modifying selected parameters in a workflow.

Several gaps still exist in the current version of the package. First, the processing of remotely-sensed data implemented in the package now focuses on Landsat and Sentinel-2 datasets obtained through the Planetary Computer STAC catalogue. This focus is motivated by the ease of access to Landsat and Sentinel-2 data through this catalogue. Other EO datasets and/or data from other STAC catalogues may not necessarily be processed with the current implementation of the functions. However, the structure of the function should make it straightforward to implement processing from additional STAC catalogues.

A second consideration is that, in the current version of the control-impact matching, it is assumed *a priori* that all the evaluated units have values for all covariates. This may not always be the case, for example, for datasets that contain spatial and/or temporal data gaps. In such cases, an error will occur or impact/control units may be excluded for matching. Future version of the package should consider detection of missing data and returning of warning messages or implementation of matching methods that can handle missing data.

The calculation of the p-value for significance of the BACI contrast is subject to the general assumptions made in the t-test of independence, normality and homogeneity of variance. The functions in the package do not test these assumptions and the p-values should, therefore, be used with caution.

Finally, while we tried to implement efficient download and processing of EO through the use of data cubes that allow lazy processing, the processing of large datasets can still require significant and potentially prohibitive processing times. Currently, no interface for cloud native processing infrastructure is provided. Further development of the package could include the possibility to link to EO cloud processing back-ends.

## Web location (URIs) and repository

The package is available on GitHub: github.com/Space4Restoration/spatialBACI. Package documentation and tutorials can be found at space4restoration.github.io/spatialBACI/.

## Usage rights

The software is distributed under the GNU General Public License (version 3 or later).
